# Supplementary material for: Synthetic biodegradable microporous hydrogels for in vitro 3D culture of functional human bone cell networks
Source: Nat Commun. 2024 Jun 13;15:5027. doi: 10.1038/s41467-024-49280-3 (PMC11176307; doi:10.1038/s41467-024-49280-3)
Supplement: Supplementary file 1 — Supplementary Information [file 41467_2024_49280_MOESM1_ESM.pdf]

Supplementary Information

Synthetic Biodegradable Microporous Hydrogels for *In Vitro* 3D Culture of Functional Human Bone Cell Networks

Doris Zauchner[1], Monica Zippora Müller[1], Marion Horrer[1], Leana Bissig[1], Feihu Zhao[2], Philipp Fisch,[1] Sung Sik Lee[3], Marcy Zenobi-Wong,[1] Ralph Müller[1] & Xiao-Hua Qin[1]

---

[1]Institute for Biomechanics, ETH Zürich, Zürich, Switzerland.

[2]Department of Biomedical Engineering and Zienkiewicz Centre for Computational Engineering, Swansea University, Swansea, United Kingdom.

[3]Institute of Biochemistry and Scientific Center of Optical and Electron Microscopy, ETH Zürich, Zürich, Switzerland.

Correspondence should be addressed to X.Q. (email: qinx@ethz.ch)

Supplementary Methods

Synthesis of 4-arm poly(ethylene glycol) vinylsulfone (4-PEG-VS): Rhodamine-labeled 4-PEG-VS and 4-PEG-VS were synthesized according to the protocol by Broguiere et al.[1] In a 2-neck flask, 4 mL of triethanolamine (TEOA, Sigma-Aldrich, 90279- 100ML) buffer (200 mM, pH 8.0) was purged with argon for 15 min to remove oxygen. Then, 1 g (50 µmol) of 20 kDa 4-PEG-thiol (Laysan Bio, SH-20K-1g) was added and stirred under argon protection. After dissolution, the solution was carefully transferred into a syringe and immediately added dropwise (1 drop every 3 s) over vigorous stirring to 1.05 mL (10 mmol) of divinyl sulfone (97%, Alfa Aesar, L12827-09) in 4 mL of the same buffer. The reaction was left to proceed for 2 h. The product was dialyzed (M_W cut-off: 3.5 kDa) against ultrapure water with 6 changes of water over at least 4 h each, sterile filtered, and lyophilized after removal of excessive water using a rotary evaporator (Heidolph, 55 mbar, 200 rpm, 40°C). The product was aliquoted and stored at -20°C until use.

Synthesis of Rhodamine-labeled 4-PEG-VS: In a flask, 2 mL of ultrapure water was protected with argon for 15 min. Then, 200 mg (10 µmol) of 20 kDa 4-PEG-thiol was dissolved in it. To this flask, a solution containing 0.24 mg (0.5 µmol) of tetramethyl rhodamine-5-maleimide (Sigma-Aldrich, 94506) in 2 mL phosphate buffered saline (PBS, Gibco, 10010-015, pH 7.4) was added dropwise over stirring. The conjugation happened within seconds but was left to proceed for 10 minutes. The resulting mixture was added dropwise into 462.5 µL (4.4 mmol) of divinyl sulfone in 4 mL TEOA buffer (200 mM, pH 8.0), left to react for 60 min under stirring, dialyzed, sterile filtered, aliquoted, and lyophilized. All the handling was performed in the dark. This protocol substitutes 1/80th of the 4-arm-PEG ends in the upper limit case of 100% conjugation efficiency.

Time-Lapsed Pore Imaging: For time-lapsed imaging of polymerization-induced phase separation (PIPS), rhodamine-labeled 4-PEG-VS was used when preparing the hydrogel precursor solution to image the 4-PEG-VS phase in the hydrogel. The hydrogel was crosslinked directly in a custom-made poly(dimethylsiloxane) (PDMS) mold inside a confocal microscope (Leica SP8) at 37°C. Using a 63× oil immersion objective with 1.4× zoom, z-stacks of 35 µm were obtained every 2 min for 90 min.

Rheology: For rheology, acellular hydrogel precursor solutions with varying compositions were prepared as described above and analyzed on an Anton Paar rheometer MCR302 (82868246) using a PP20 plate and a glass bottom. Hydrogels were crosslinked at 37°C for 60 min while a time-sweep measurement was performed at 1 Hz, 5% strain and with a gap of 100 µm to determine the hydrogel storage modulus (*G′*). For each sample, 40–50 µL hydrogel solution was loaded into the center of the glass plate and after setting the PP20 plate to the desired gap position, mineral oil (Sigma-Aldrich, 330779) was placed around the hydrogel to prevent dehydration. Frequency sweep was performed to test time-dependent viscoelastic properties of the hydrogels (oscillatory strain: 5%, angular frequency: 0.1-100 rad s^{-1}, 37°C). Stress relaxation tests were performed using a sand-blasted PP20 plate to avoid slipping of the hydrogel during the measurement. Samples of 100 µL hydrogel precursor were crosslinked for 60 min at 37°C surrounded by silicone oil to prevent dehydration while applying 5% oscillatory strain at 1 Hz. Subsequently, the strain was kept constant at 5% for 6 h while monitoring the shear stress. For each sample, the shear stress was normalized to the initial value measured at 5% constant strain. The stress relaxation time $t_{0.6}$ is the time at which the stress reached 60% of the initial value.

Human Mesenchymal Stromal Cell (hMSC) Culture: For 2D cell expansion, hMSC (Lonza, PT-2501) were cultured in expansion medium containing Dulbecco's modified Eagle's medium (DMEM, Gibco, high glucose) with 10% v/v fetal bovine serum (FBS, Gibco, Lot#42F7190K or 2440094), 1% v/v Antibiotic-Antimycotic (Anti-Anti, Gibco, 15240-062), 1% v/v non-essential amino acids (Gibco, 11140-035) and 1 ng mL^{-1} basic fibroblast growth factor (Invitrogen, 13256-029) in T150 cell culture flasks (TPP, 90151) at 37°C with 5% CO_2 until reaching 80% confluency. Medium was exchanged 3 times per week. Cells were washed twice with PBS (37°C) before adding 0.25% trypsin-EDTA (Gibco, 25200-056) to detach them from the flask. Trypsin activity was blocked by adding control medium (DMEM + 10% v/v FBS + 1% v/v Anti-Anti). Cells were counted using a hemocytometer and resuspended in control medium at the desired concentration. hMSC (p5–p8) were embedded inside microporous hydrogels by resuspending them in the hyaluronic acid (HA) stock solution during hydrogel preparation to obtain final cell concentrations of 5×10^5–5×10^6 mL^{-1}. They were subsequently cultured in osteogenic differentiation medium (control medium with 10 mM β- glycerophosphate (Acros, 410991000), 50 µg mL^{-1} L-ascorbic acid (Sigma-Aldrich, A92902-100G) and 100 nM dexamethasone (Sigma-Aldrich, D2915)). Medium was replaced 3 times a week in custom PDMS molds and 5 times a week for static culture on chip. For the preliminary dynamic cell culture, fluid shear stress (FSS) was applied on chip (AIM Biotech, DAX-1) by connecting two syringe pumps to both inlets of one medium channel and applying a total flow rate of 20 µL min^{-1} (low FSS) or 200 µL min^{-1} (high FSS) using DMEM. Loading was performed 2×10 min daily starting on day 3 until day 7 of culture and then again from day 10 until day 13 with 60 min in between each treatment. Three replicates were used per condition (static, low FSS, high FSS). An additional dynamic cell culture was performed using a 24-channel peristaltic pump (Longer, BT100-1L) to apply a flow rate of 438 µL min-1 (corresponding to a FSS τa=2 Pa, as simulated by the CFD model) to dynamic samples. Perfusion was performed using DMEM supplemented with 1% Anti-Anti for 10 min 3 times per week for up to 21 days. Static controls were cultured on chip, and osteogenic medium was exchanged 3 times per week.

Human Osteoblast (hOB) Culture: Primary hOB were obtained from a commercial supplier (PromoCell, C-12720) or from the University Children's Hospital Zurich under ethical approval (KEK-ZH-Nr. 2019-00811) from healthy donors. For 2D cell expansion, hOB (p5–p6) were cultured similarly as hMSC until reaching 80% confluency. After embedding in PEG hydrogels, hOB were cultured in the same osteogenic differentiation medium as hMSC for 2 days before fixation.

Live/Dead Assay: To quantify cell viability, staining with Calcein Green AM (CaAM, Sigma-Aldrich, 56436-50UG) and Ethidium-homodimer-1 (EthD-1, Sigma-Aldrich, 460439) was performed. Staining solution (1:1000 EthD-1 and 1:500 CaAM in PBS) was applied after washing samples twice with PBS and then incubated for 15–20 min at 37°C protected from light before washing again with PBS. Samples were imaged using confocal microscopy (Leica SP8) with a 10× air objective. For analysis, maximum intensity projections (MIP) of z-stacks of 70–100 µm each were created in Fiji/ImageJ. Cells in green and red channel were either counted manually if discrimination between single cells was not possible or a custom-written macro was used. Viability was then calculated as the percentage of live cells among all present cells in the MIP.

Fixation and F-Actin-Nuclei Staining: At the end of culture, cells were fixed by first washing them with PBS and then applying a solution of 4% paraformaldehyde (Sigma-Aldrich, 15-812-7) for 15–20 min at room temperature. Samples were washed twice with PBS. F-Actin-nuclei staining was performed to further investigate cellular morphology and cell network formation. Hydrogels were incubated in 1% v/v bovine serum albumin (BSA, Sigma-Aldrich, 9048-46-8) in PBS for 1.5 h at room

temperature. Subsequently, cells were permeabilized in a solution of 0.2% w/v Triton X-100 (Sigma-Aldrich, 9002-93-1) in 0.1% BSA in PBS for 10 min. Hydrogels were washed 3 times with PBS. The staining solution containing dilutions of 1:1000 Hoechst 33342 (1:1000, Sigma-Aldrich, B226) and 1:200 Phalloidin CruzFluor 647 Conjugate (Santa Cruz Biotechnology, sc-363797) or Phalloidin-TRITC (Sigma-Aldrich, P1951) in 0.1% BSA was prepared. On-chip samples were stained for 12–24 h at 4°C, samples in confocal dishes for 1.5–2.0 h at room temperature protected from light. Before image acquisition, on-chip samples were washed 5 times with 5 min between each wash and hydrogels on confocal dishes were washed 3 times. Imaging was performed using confocal microscopy (Leica SP8).

Cryosectioning: Prior to cryosectioning, fixed samples were cryoprotected overnight in 30% w/v sucrose (Sigma-Aldrich, S7903) in PBS at 4°C. The next day, the samples were soaked in 1:1 sucrose (30%) and optimal cutting temperature (OCT) compound (Tissue-Tek) solution for 4 h. Samples were then transferred into a cryomold (Tissue-Tek, 25×20×5 mm), covered in OCT and frozen in liquid nitrogen. Using a histology cryotome (Thermo Fisher, CryoStar NX70), sections of 10–40 µm were cut.

Alizarin Red Staining: To assess matrix mineralization in cryosections and within microfluidic chips, calcium deposits were stained with Alizarin red. A staining solution (2 mg mL^{-1} Alizarin red S (Sigma-Aldrich, A5533-25G) in distilled water, pH adjusted to 4.12) was applied to the samples after two washes with distilled water. The samples were stained for 30 min at room temperature and then washed 5 times until the water came out clear. Imaging at 5–20× magnification was performed on Leica DMi1 microscope. A color threshold was applied by selecting only the red channel in RGB color space in Fiji/ImageJ. The area of red color was then measured for each condition.

Osteocalcin Staining of Cryosections: For osteocalcin immunostaining of cryosections, non-specific antibody binding was first blocked with 1% BSA w/v and 5% serum v/v from the host of the secondary antibody for 1 h (goat serum). Primary antibodies were diluted in PBS containing 1% BSA. Immunostaining of osteocalcin was performed using Anti-Osteocalcin (1:200, rabbit polyclonal, Abcam, ab93876) overnight at 4°C. Samples were washed 3×5 min with PBS before incubation with the secondary antibody (1:500, goat anti-rabbit IgG H&L Alexa 555) for 1 h. Immunofluorescence staining was validated by a secondary antibody control without adding the primary antibody. Samples were mounted with Mowiol. Sections were imaged using confocal microscopy with a 63× oil immersion objective. To quantify osteocalcin expression, the fluorescence intensity per image was measured as integrated density in Fiji/ImageJ followed by normalization by the cell number in each image.

Alkaline phosphatase (ALP) assay: The enzymatic activity of ALP in cells within PEG hydrogels was assessed using a colorimetric assay based on the enzyme's ability to convert p-nitrophenylphosphate into p- nitrophenol. PEG hydrogels containing 10^5 cells each were washed with PBS. Subsequently, 0.5 mL of 0.2% w/v Triton X-100 with 5 mM MgCl2 was added to each hydrogel in an Eppendorf tube, followed by a 30-minute incubation period. Hydrogels were then homogenized using Fisherbrand™ Pellet Pestle™, and the homogenized samples were centrifuged at 3000 g for 10 min. Following centrifugation, 200 µL of the supernatant was collected in new Eppendorf tubes. For the ALP assay, the following reagents were pipetted into a clear flat-bottom 96-well plate: 80 µL of the sample or standard, 20 µL of 0.75 M 2-amino-2-methyl-1-propanol (AMP, pH adjusted to 10.5, Sigma, A65182) and 100 µL of substrate solution. The substrate solution was prepared by mixing 37.11 mg of p-nitrophenylphosphate disodium salt hexahydrate (Sigma, 71768-5G) with 1 mL of 1.5 M AMP buffer and 9 mL of ultrapure water (UPW). P-nitrophenol standards (Sigma-Aldrich, 425753) were prepared from a 1 mM stock solution in 0.2% w/v Triton X-100 with 5 mM $MgCl_2$ and diluted to concentrations

of 0, 0.05, 0.2, 0.6 and 0.9 µmol mL^{-1}. The reaction was left to proceed for 15 min before 100 µL 0.2 M NaOH was added to stop the reaction. The absorbance at 405 nm was then measured on a plate reader (Tecan, Spark 10M). Samples and standards were measured in technical duplicate and averaged, and the absorbance of the zero standard was subtracted from all samples and standards before the concentration of p-nitrophenol was calculated from the standard curve. The obtained concentrations were normalized by the reaction time (15 min) and finally by the DNA content in each sample as detailed below.

DNA assay: After completing the ALP assay, the remaining volume of homogenized hydrogels underwent three cycles of freeze-thawing and ultrasonication. Subsequently, the samples were incubated at room temperature for 48 hours. DNA quantification was performed using the Quant-iT PicoGreen dsDNA assay kit (Invitrogen, P7589) according to the manufacturer's instructions. In short, 87.5 µL of 1×TE buffer, 12.5 µL of the sample and 100 µL of PicoGreen working solution were added to each well of a clear, flat-bottom 96-well plate. Standards were prepared according to the manufacturer's protocol. Samples and standards were measured in duplicate and the results averaged. After 5 min of incubation in the dark, fluorescence emission at 535 nm with an excitation at 485 nm was measured using the plate reader. For quantification, the zero standard was subtracted from all measurements and the sample DNA concentration was calculated from the standard curve.

RNA Isolation and Reverse Transcription: PEG hydrogels with hMSC (~10^5 cells per sample) were cultivated for 0, 7, 14 or 21 days in osteogenic medium, washed with PBS and shock frozen in liquid N_2. Samples were stored at -80°C until further use. Hydrogels were thawed and homogenized in 300 µL Trizol (Invitrogen, 15596018) with a Fisherbrand™ Pellet Pestle™ and a 21G needle. 700 µL Trizol were added to each sample and samples were incubated for 5 min at room temperature. Samples were centrifuged for 30 s at 8500 g and the supernatant was collected. 200 µL Chloroform (Sigma-Aldrich, C2432-500ML) were added before vortexing. After incubating for 5 min, samples were centrifuged for 20 min at 12000 g at 4°C. The aqueous phase was collected and 1 volume of 70% EtOH was added. The samples were then transferred on RNeasy MinElute spin columns and the manufacturer's protocol of the RNeasy Micro kit (Qiagen, 74004) was followed with a few exceptions: Washing steps 4, 6 and 7 were repeated twice each and RNA was eluted in 28 µL H_2O. 20–22 µL RNA were reversed transcribed using iScript™ Reverse Transcription Supermix for RT-qPCR (Bio-Rad, 1708840) according to the manufacturer's protocol. After synthesis, the cDNA was diluted 1:5 in ultrapure H_2O.

Quantitative PCR (qPCR): 7.2 µL of cDNA were mixed with 8 µL TaqMan™ Fast Universal PCR Master Mix (2X), no AmpErase™ UNG (Applied Biosystems, 4366072) and 0.8 µL TaqMan™ Gene Expression Assay Primers (Supplementary Table 2). A dilution series of 1v:1v (1, 1/2, 1/4, 1/8, 1/16) of a standard sample was used for relative quantification of the samples. Samples were initially heated 30 s at 95°C followed by up to 50 cycles of 5 s at 95°C and 20 s at 60°C in a CFX96™ Real-Time System C1000 Touch™ Thermal Cycler. Samples were analyzed in technical duplicate. Gene expression data was normalized to the housekeeping gene β-actin (*ACTB*).

Immunofluorescence Staining of 3D Hydrogels: To investigate the expression of osteogenic markers in 3D hydrogels in molds and on chip, fixed hydrogels were first incubated in 0.3% w/v Triton X-100 in PBS for 20 min. Non-specific antibody binding was then blocked with 1% BSA w/v and 5% serum v/v from the host of the secondary antibody for 45 min (donkey serum, Abcam, ab7475). Primary antibodies, including Anti-Osteocalcin (rabbit polyclonal, Abcam, ab93876), Anti-Collagen I

(mouse monoclonal, Abcam, ab6308), and Anti-Podoplanin (mouse monoclonal, Santa Cruz Biotechnology, sc-59347), were diluted to a 1:200 concentration in the same blocking buffer. Samples were then incubated overnight at 4°C in this solution. To serve as a negative control, primary antibodies were omitted in selected samples. Following primary antibody incubation, samples were washed 3× 5 min in 0.025% w/v Triton X-100 in PBS. Corresponding secondary antibodies (donkey anti-mouse IgG AF488, A21202 and donkey anti-rabbit IgG AF 647, A-31573) were diluted to 1:500 in 1% BSA in 0.3% Triton X-100 in PBS each. To counterstain the cytoskeleton and nuclei, phalloidin-TRITC and Hoechst 33342, respectively, were added at a dilution of 1:500. Samples were incubated in the secondary antibody solution for 2 h protected from light before washing them 3×5 min in 0.025% w/v Triton X-100 in PBS. 100 µm thick z-stacks were acquired using a 20× air objective on a Zeiss LSM 780 confocal microscope. To quantify osteogenic marker expression, the fluorescence intensity per image was measured as integrated density in Fiji/ImageJ followed by normalization by the cell number in each image.

Quantification of Permeability: In order to quantify the permeability of the microporous PEG hydrogels, the method described by Moreno-Arotzena et al.[2] was adapted. Acellular PEG hydrogels and collagen type I hydrogels were used. 2 mg mL^{-1} collagen type I hydrogel was prepared from an 8.91 mg mL^{-1} stock solution (rat-tail, Corning, 354249) as described by Shin et al.[3] Hydrogels were casted into the channel of a µ-Slide I Luer (Ibidi, channel height: 0.4 mm). To determine the permeability, all hydrogels were first hydrated in PBS for 24 h after crosslinking. Medium reservoirs were filled with 60 µL DMEM and 1 mL syringe barrels without plunger were attached to the luer connectors. 0.5 mL DMEM were added to one of the barrels and 0.1 mL to the other one creating a height and pressure difference that caused interstitial flow through the porous hydrogel. The heights h_1 and h_2 were measured to calculate the difference in height h_d. From this, the pressure difference ΔP was calculated using **Equation 1**, where g is the gravitational acceleration and ρ is the density of the medium (ρ_{DMEM}=1000 kg m^{-3}).

$$\Delta P=\rho\times g\times h_d \tag{1}$$

The change of height was measured every 15 min for the first hour and then every 30 min for 4 h in total. An exponential function was fitted to the data according to $\Delta P(t)=P(0)\times e^{-ct}$ to determine the exponent c. Since ΔP changes rapidly within the first hour, only data from time-points 0.75–4 h was considered. Permeability K and the constant c are related according to Darcy's law in **Equation 2**, where μ is the viscosity of DMEM (7.8×10^{-4} Pa s), l is the length of the hydrogel channel (1.70×10^{-2} m), A_r is the cross section of the syringe barrel (9.62 mm^2) and A is the cross section of the hydrogel channel in the direction of fluid flow (1.52 mm^2).

$$K=\frac{c\times\mu\times l\times A_r}{\rho\times g\times A} \tag{2}$$

Computational Fluid Dynamics (CFD) Simulation: Confocal images of rhodamine-labeled PEG hydrogel on chip (AIM Biotech, DAX-1) were processed, and the pore geometry was reconstructed using Seg3D (University of Utah, UT, USA). To quantify the FSS within the scaffold that has highly irregular porous geometries, a multiscale and multiphase CFD model previously developed[4] was used. The model involves 2 scales, i.e. (i) the global scale that represents the whole microfluidic chip and (ii) the local scale that models the detailed micro-structures of subsections (dimension: 20×20×30 µm, n=4) from the whole scaffold. In the global model, the scaffold region was modelled as porous media with a permeability of 8.67×10^{-15} m^2, which was obtained from experimental measurement of a hydrogel with a composition containing 2.2% 4-PEG-VS and 1.0% 40 kDa dextran matching the confocal

microscopy data. To approximate the experimental condition for dynamic cell culture, two types of flow rates (i.e., 10 μL min^{-1} and 100 μL min^{-1} per port) were applied to the global model as inlet and outlet boundary conditions. Mass flux conservation was applied to the interface between porous media and free fluid. The global model was meshed with 450410 tetrahedral elements. The pressure gradient that was calculated from the global model was applied to the local CFD model for simulating the shear stress on PEG scaffold surfaces. The boundary conditions of local CFD model are shown in Supplementary Figure 16. In the local model, the fluid domain of each subsection was meshed by a uniform tetrahedral element size of 0.4 μm, which generated 1322072, 1135226, 1247121, and 1302269 elements, respectively, for 4 discretized subsections. In this study, the fluid was modelled as laminar flow with the dynamic viscosity of DMEM (7.8×10^{-4} Pa s). The CFD models were solved by a finite volume method (FVM) using ANSYS CFX (ANSYS Inc., PA, USA) under the convergence criteria of root-mean-square residual of the mass and momentum $<10^{-4}$. The same methods for (i) confocal images – based geometry reconstruction and (ii) CFD model setup were applied to the PEG hydrogel scaffold with 500 kDa dextran with a permeability of 2.97×10^{-13} m^2. In the local model, 4 subsections were meshed with 1664388, 1790433, 1398725 and 1449911 elements, respectively. The varying flow rates of 100 – 1000 μL/min were applied to the inlets of the chip (global model), under which the corresponding FSS within each subsection was computed. Then the optimal flow rate was recorded for the desirable average FSS.

Supplementary Figures

Figure	4-PEG-VS [%]	crosslinker	SH/ene	dextran [%]	dextran M_w	HA [%]	RGD	cell density [mL^{-1}]
**2b**	2.5 (labeled)	PEG-2-SH 2.0 kDa	0.80	1.0	40	0.50	GRCGRGDSPG	-
**2c**	2.0–2.5	KCGPQGIWGQCK, PEG-2-SH 2.0 kDa	0.80	1.0	40	0.50	GRCGRGDSPG	-
**2d**	2.0	GCRDGPQGIWGQDRCG, PEG-2-SH 3.4 kDa	1.25 (MMP), 0.80 (PEG-2-SH)	0.0–1.0	500	0.50	CGRGDSP	-
**2e**	2.0	GCRDGPQGIWGQDRCG, PEG-2-SH 3.4 kDa	1.25 (MMP), 0.80 (PEG-2-SH)	1.0	500	0.50	-	-
**3a-c**	2.0 (labeled)	KCGPQGIWGQCK	0.80	0.5–2.0	40	0.50	-	-
**3d–f**	2.0 (labeled)	KCGPQGIWGQCK	0.80	1.0	40, 500	0.50	-	-
**4b**	2.0	GCRDGPQGIWGQDRCG, PEG-2-SH 2.0 kDa	0.80	1.0	40	0.50	GRCGRGDSPG	$5.0{\times}10^5$
**4c**	2.0	GCRDGPQGIWGQDRCG, PEG-2-SH 2.0 kDa	0.80	1.0	40	0.50	GRCGRGDSPG	$5.0{\times}10^5$
**4d**	2.0	GCRDGPQGIWGQDRCG, PEG-2-SH 2.0 kDa	0.80	1.0	40	0.50	GRCGRGDSPG	$3.5{\times}10^6$
**4e–h**	2.0	GCRDGPQGIWGQDRCG, PEG-2-SH 3.4 kDa	1.25 (MMP), 0.80 (PEG-2-SH)	1.0	500	0.50	CGRGDSP	$3.0{\times}10^6$
**5a–g**	2.0	GCRDGPQGIWGQDRCG, PEG-2-SH 3.4 kDa	1.25 (MMP), 0.80 (PEG-2-SH)	1.0	500	0.50	CGRGDSP	$3.0{\times}10^6$
**6a–e**	2.0	GCRDGPQGIWGQDRCG, PEG-2-SH 2.0 kDa	0.80	1.0	40	0.50	GRCGRGDSPG	$3.5{\times}10^6$
**7a,b**	2.2	KCGPQGIWGQCK	0.80	1.0	40	0.50	GRCGRGDSPG	-
**7c–e**	2.0	GCRDGPQGIWGQDRCG	1.25	1.0	40, 500	0.50	CGRGDSP	-
**7f,g**	2.0	GCRDGPQGIWGQDRCG	1.25	1.0	500	0.50	CGRGDSP	-
**8a**	2.0	KCGPQGIWGQCK, PEG-2-SH 3.4 kDa	0.80	1.0	500	0.50	CGRGDSP	$3.0{\times}10^6$
**8b**	2.2	KCGPQGIWGQCK	0.80	1.0	40	0.50	CGRGDSP	$5.0{\times}10^5$, $1.0{\times}10^6$
**8c–e**	2.0	GCRDGPQGIWGQDRCG	1.25	1.0	500	0.50	CGRGDSP	$3.0{\times}10^6$
**SF1**	2.0 (labeled)	PEG-2-SH 2.0 kDa	0.80	0, 1	40	0.50	-	-
**SF2a**	2.0–2.5	KCGPQGIWGQCK, PEG-2-SH 2.0 kDa	0.80	1.0	40	0.50	-	-
**SF2b**	2.0	PEG-2-SH 2.0 kDa	0.80	1.0	40, 500	0.50	-	-
**SF2c**	2.5	PEG-2-SH 2.0 kDa	0.80	1.0	40	0.25–0.83	-	-
**SF2d**	2.0	KCGPQGIWGQCK, PEG-2-SH 2.0 kDa	0.80	1.0	40	0.50	CGRGDSP	-
**SF3**	2.0 (labeled)	KCGPQGIWGQCK	0.80	0.5–2.0	40	0.50	-	-
**SF4**	2.0 (labeled)	KCGPQGIWGQCK	0.80	1.0	40, 500	0.50	-	-
**SF5**	2.0 (labeled)	KCGPQGIWGQCK	0.80	1.0	40, 500	0.50	-	-
**SF6**	2.0	KCGPQGIWGQCK, PEG-2-SH 2.0 kDa	0.80	1.0	40	0.50	CGRGDSP	$2.0{\times}10^6$
**SF7**	2.0	GCRDGPQGIWGQDRCG, PEG-2-SH 2.0 kDa	0.80	1.0	40	0.50	GRCGRGDSPG	$3.5{\times}10^6$
**SF8**	2.0	GCRDGPQGIWGQDRCG, PEG-2-SH 3.4 kDa	1.25 (MMP), 0.80 (PEG-2-SH)	1.0	500	0.50	CGRGDSP	$3.0{\times}10^6$
**SF9**	2.0	GCRDGPQGIWGQDRCG, PEG-2-SH 3.4 kDa	1.25 (MMP), 0.80 (PEG-2-SH)	1.0	500	0.50	CGRGDSP, -	$3.0{\times}10^6$
**SF10**	2.0	GCRDGPQGIWGQDRCG	1.25	0.2, 1.0	500	0.50	CGRGDSP	$3.0{\times}10^6$
**SF11**	2.0	GCRDGPQGIWGQDRCG, PEG-2-SH 3.4 kDa	1.25 (MMP), 0.80 (PEG-2-SH)	1.0	500	0.50	CGRGDSP	$3.0{\times}10^6$
**SF12**	2.0	GCRDGPQGIWGQDRCG, PEG-2-SH 3.4 kDa	1.25 (MMP), 0.80 (PEG-2-SH)	1.0	500	0.50	CGRGDSP	$3.0{\times}10^6$
**SF13**	2.0	GCRDGPQGIWGQDRCG	1.25	1.0	500	0.50	CGRGDSP	$3.0{\times}10^6$
**SF14**	2.0	GCRDGPQGIWGQDRCG, PEG-2-SH 2.0 kDa	0.80	1.0	40	0.50	GRCGRGDSPG	$3.5{\times}10^6$
**SF15**	2.2	KCGPQGIWGQCK	0.80	1.0	40	0.50	GRCGRGDSPG	-
**SF17**	2.2	KCGPQGIWGQCK	0.80	1.0	40	0.50	CGRGDSP	-
**SF18**	2.2	KCGPQGIWGQCK	0.80	1.0	40	0.50	CGRGDSP	$1.0{\times}10^6$
**SF19**	2.0	GCRDGPQGIWGQDRCG	1.25	1.0	500	0.50	CGRGDSP	$3.0{\times}10^6$
**SF20**	2.0	GCRDGPQGIWGQDRCG	1.25	1.0	500	0.50	CGRGDSP	$3.0{\times}10^6$

Supplementary Table 1. Overview of PEG hydrogel compositions used in this manuscript for experiments in Figures and Supplementary Figures (SF).

**Gene**	**TaqMan ID**
Human *ACTB*	Hs01060665_g1
Human *PDPN*	Hs00366766_m1
Human *RUNX2*	Hs00231692_m1
Human *MMP14*	Hs00237119_m1
Human *ALPL*	Hs01029144_m1

Supplementary Table 2. List of primers used for qPCR.

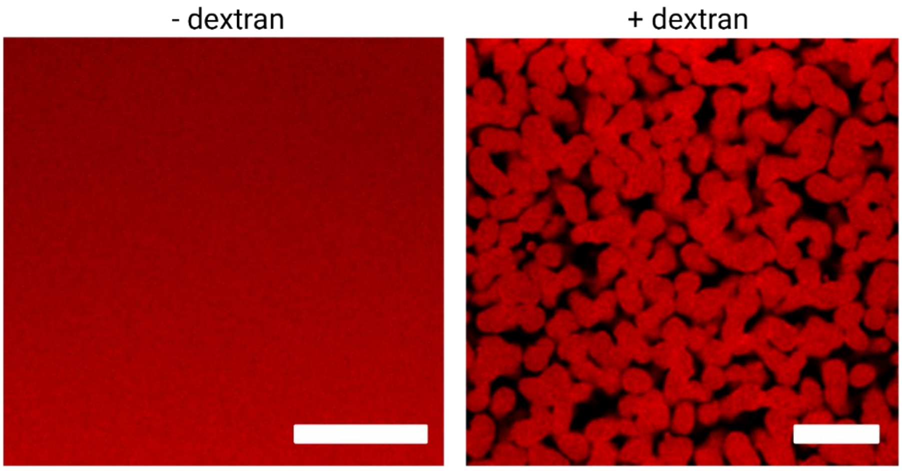

Supplementary Figure 1. Confocal microscopy images of PEG hydrogels formed by Michael addition crosslinking without and with dextran, scale bars: 10 µm.

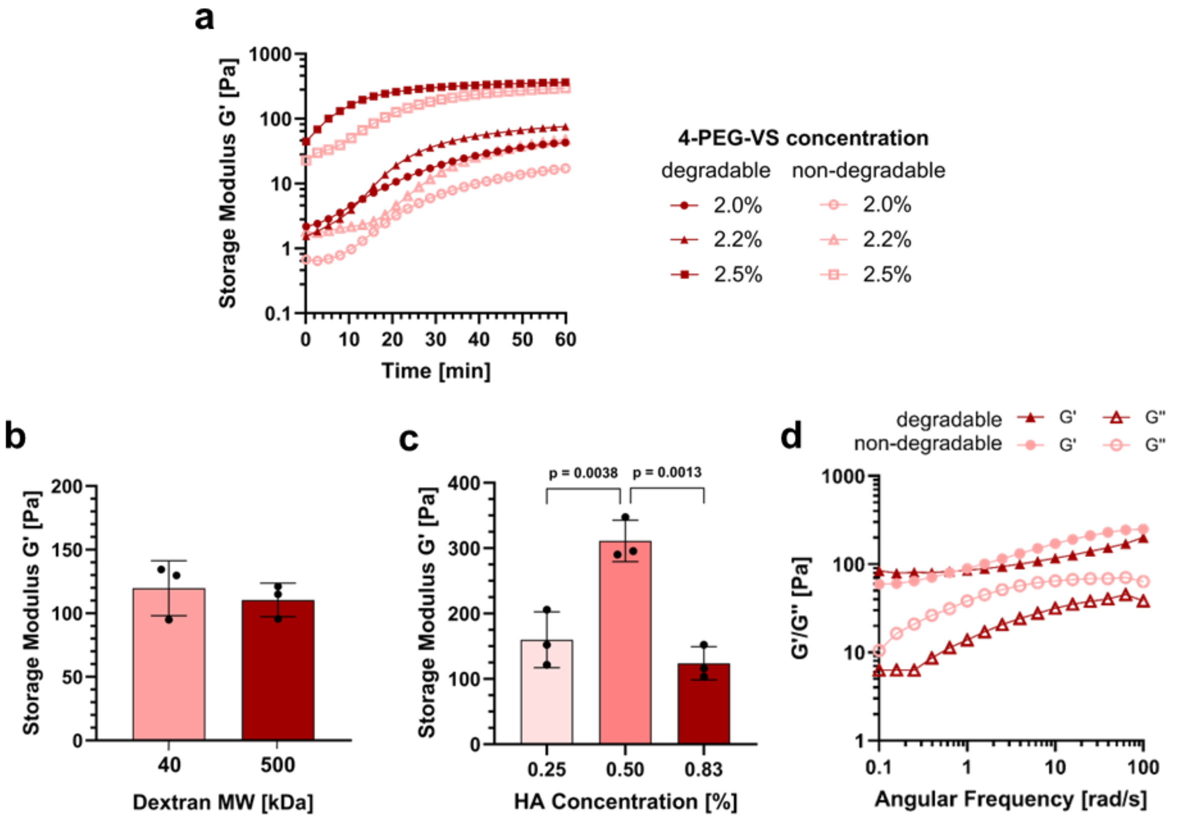

Supplementary Figure 2. Rheology of PEG hydrogels. **a)** Representative time-sweep rheology plots of PEG hydrogel compositions with varying PEG concentration during gelation at 37 ˚C (n=3 samples). **b)** Storage modulus (G') of PEG hydrogels with low (40 kDa) and high (500 kDa) dextran M_W after 60 min of crosslinking at 37 °C, n=3 samples (mean ± SD, two-sided Student's t-test). **c)** G' of PEG hydrogels with different hyaluronic acid (HA) concentration after 60 min of crosslinking at 37°C, n=3 samples (mean ± SD, one-way ANOVA/Tukey). **d)** Viscoelasticity of microporous PEG hydrogel matrices as determined by frequency sweep measurements on a rheometer at 37°C (oscillatory strain: 5%, angular frequency: 0.1–100 rad s^{-1}).

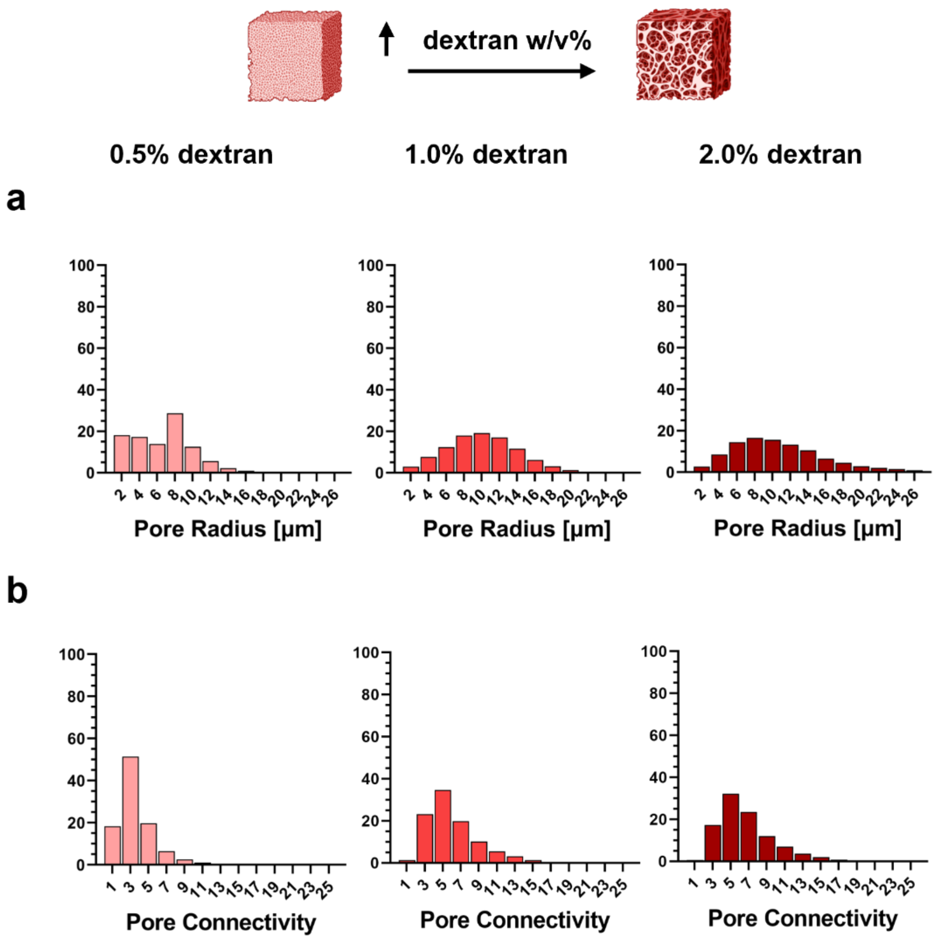

Supplementary Figure 3. Characterization of the porous architecture of void-forming PEG hydrogels with varying dextran concentration. **a)** Distribution of pore radii in PEG hydrogels with dextran concentrations ranging from 0.5–2.0%, *n*=3 samples with *n*=2 imaging positions each. **b)** Distribution of pore connectivity in PEG hydrogels with dextran concentrations ranging from 0.5–2.0%, *n*=3 samples with *n*=2 imaging positions each. Illustration, created with BioRender.com, released under a Creative Commons Attribution-NonCommercial-NoDerivs 4.0 International license.

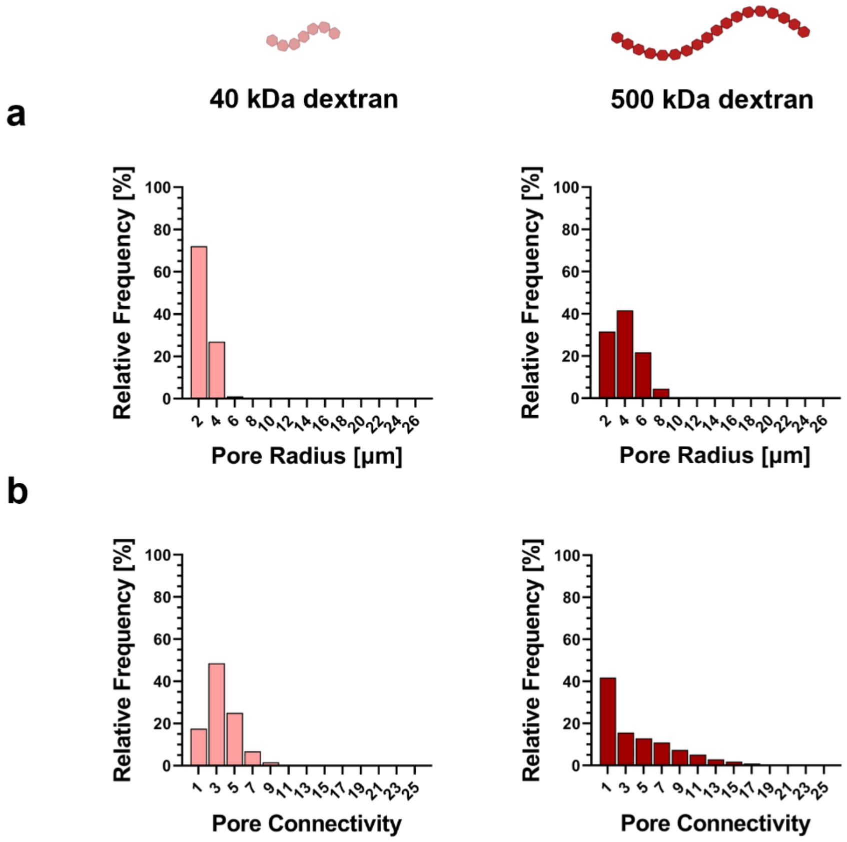

Supplementary Figure 4. Characterization of the porous architecture of void-forming PEG hydrogels with low (40 kDa) and high (500 kDa) dextran M_W. **a)** Distribution of pore radii in PEG hydrogels as a function of hydrogel composition, n=3 samples with n=3 imaging positions each. **b)** Distribution of pore connectivity in PEG hydrogels as a function of hydrogel composition, n=3 samples with n=3 imaging positions each. Illustration, created with BioRender.com, released under a Creative Commons Attribution-NonCommercial-NoDerivs 4.0 International license.

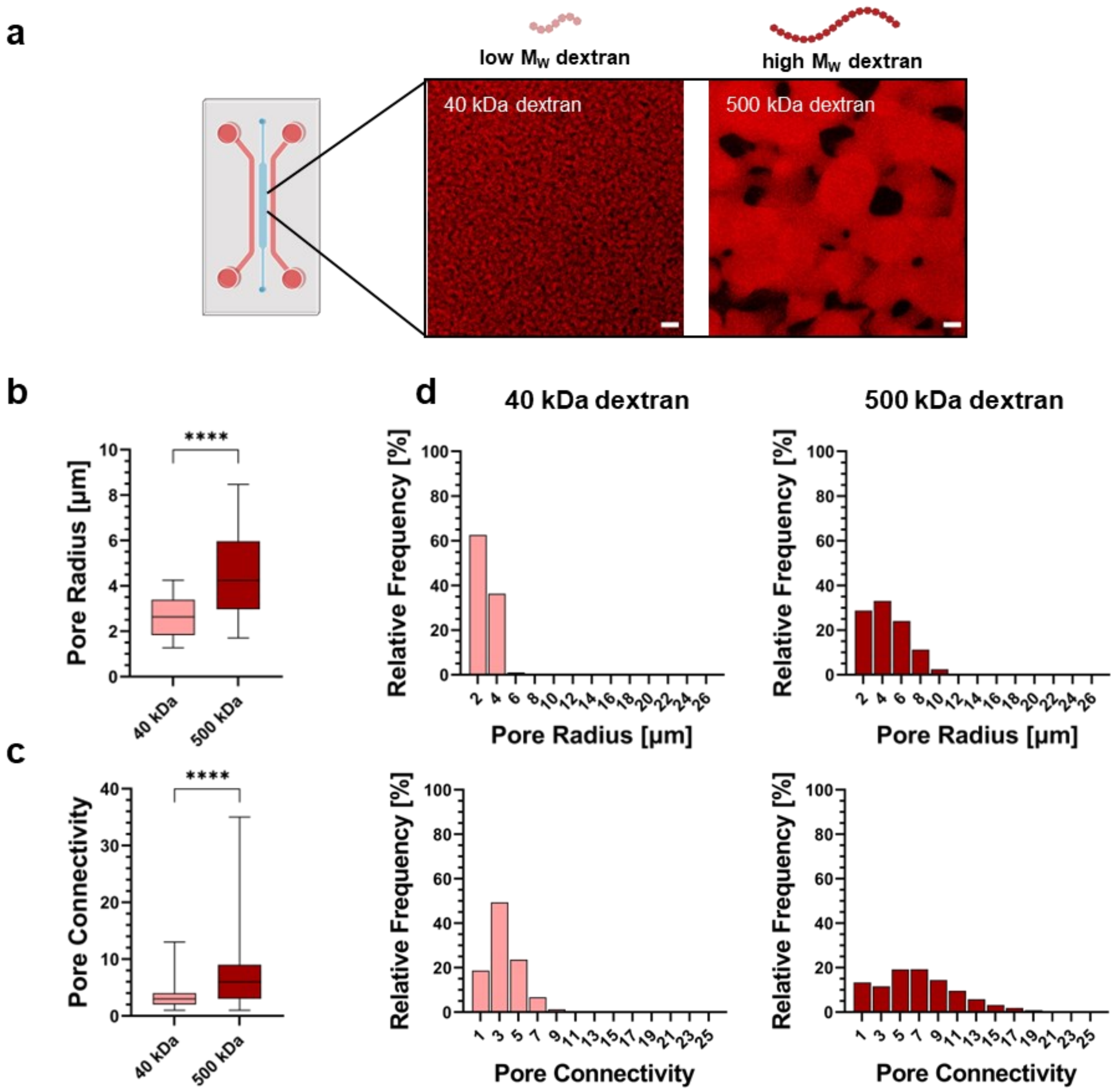

Supplementary Figure 5. Characterization of the porous architecture of void-forming PEG hydrogels on a microfluidic chip. **a)** Confocal microscopy images of rhodamine-labeled PEG hydrogels formed with 1.0% low M_W (40 kDa) and high M_W (500 kDa) dextran, scale bars: 10 µm. Illustration, created with BioRender.com, released under a Creative Commons Attribution-NonCommercial-NoDerivs 4.0 International license. **b-c)** Quantification of pore radius and pore connectivity of hydrogels formed with low M_W (40 kDa) and high M_W (500 kDa) dextran on chip, n=3 samples with n=3 imaging positions each (****p<0.0001, two-sided Student's t-test). Box plots show the 25th and 75th percentiles at the lower and upper limit, respectively, whiskers indicate minimum and maximum values, center line indicates the median. **d)** Distribution of pore radii (top) and pore connectivity (bottom) in PEG hydrogels with low and high M_W dextran on a microfluidic chip, n=3 samples with n=3 imaging positions each.

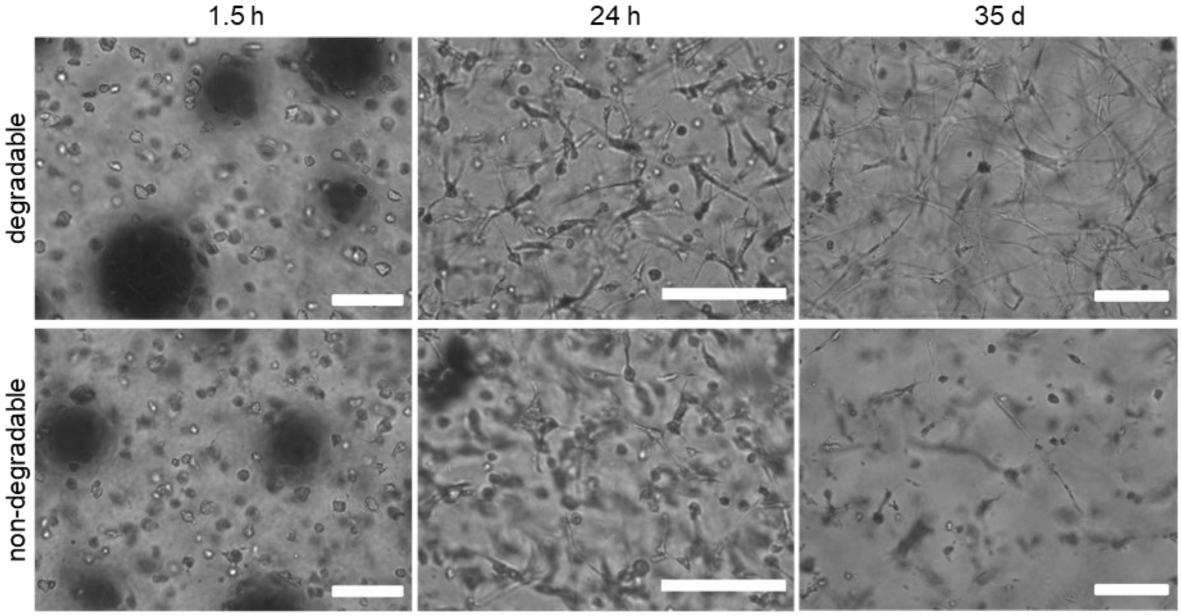

Supplementary Figure 6. Microscopy images of cell morphologies within degradable and non-degradable PEG hydrogels showing human mesenchymal stromal cell (hMSC) spreading after 1.5 h, network formation after 1 day. The cell network was stable in degradable hydrogels, whereas network degeneration was observed in non-degradable hydrogels following osteogenic cultivation for 35 days, scale bars: 50 µm.

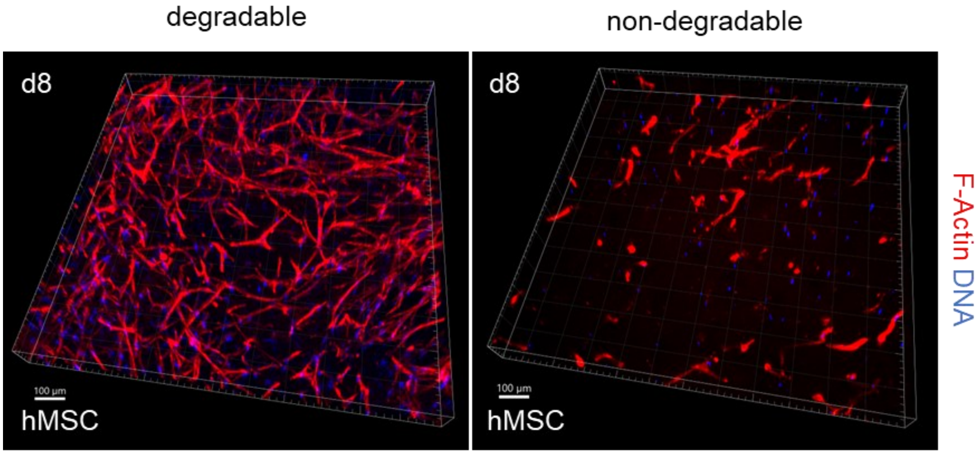

Supplementary Figure 7. Representative 3D view of actin-nuclei-stained hMSC networks in degradable and non-degradable hydrogels after 8 days of culture, scale bars: 100 µm.

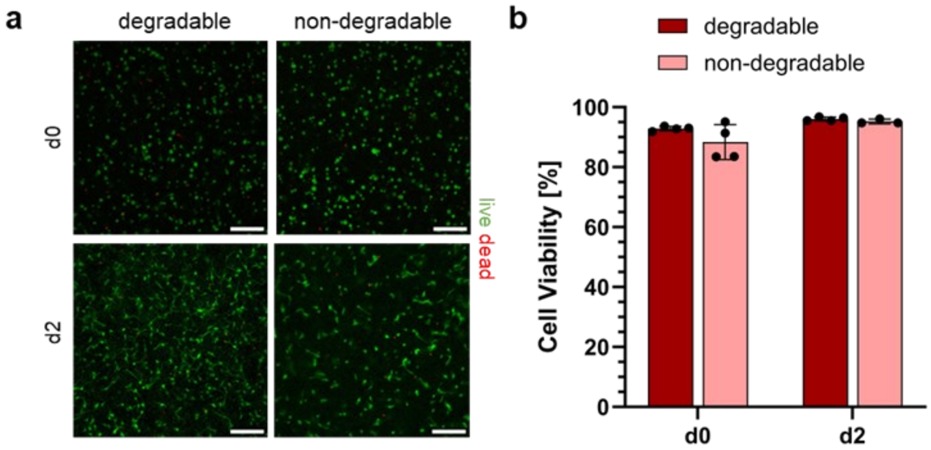

Supplementary Figure 8. Cell viability of human osteoblasts (hOB) after embedding and 2 days of osteogenic culture within degradable and non-degradable PEG hydrogels. **a)** Confocal microscopy images (MIPs) of live/dead staining, scale bars: 100 µm. **b)** Quantification of cell viability based on live/dead staining, *n*=3 samples for non-degradable d2 and *n*=4 for all other groups (mean ± SD, two-way ANOVA/Tukey).

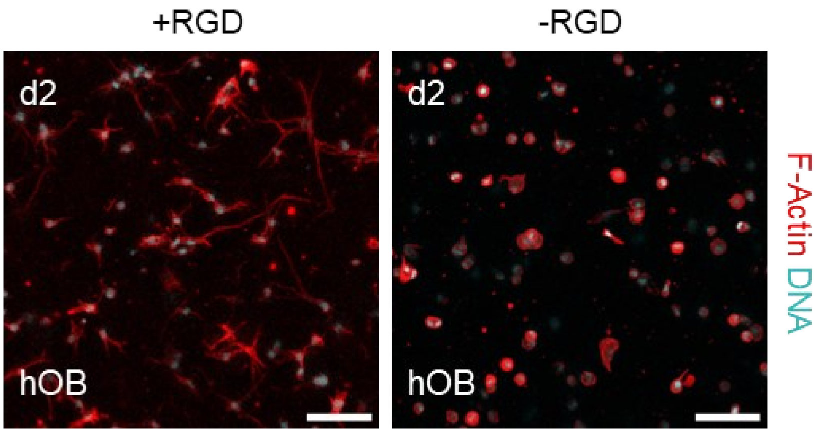

Supplementary Figure 9. Confocal microscopy images of actin-nuclei-stained hOB following 2 days of osteogenic cultivation in matrix metalloproteinase (MMP)-degradable PEG hydrogels, emphasizing the importance of RGD motifs for 3D cell network formation, scale bars: 100 µm.

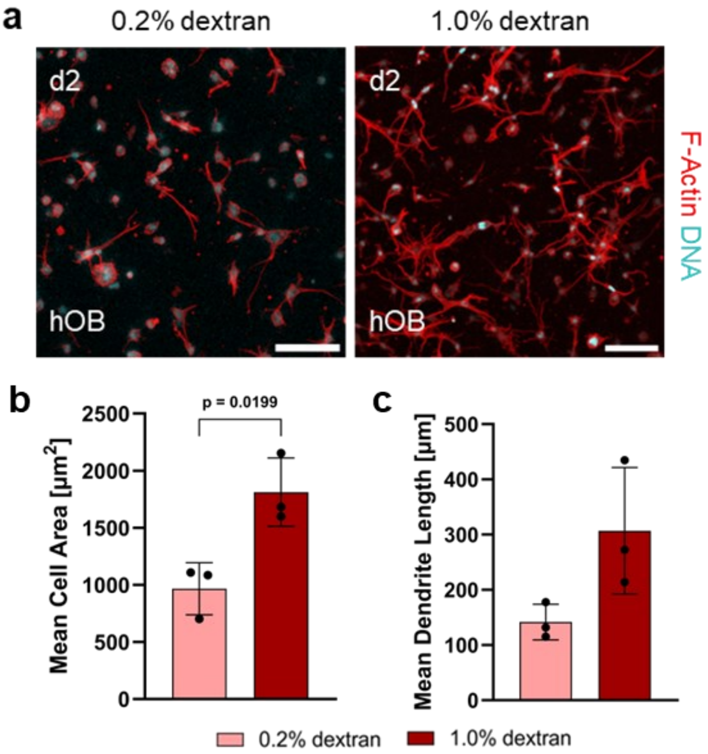

Supplementary Figure 10. a) Confocal microscopy images of actin-nuclei-stained hOB following 2 days of osteogenic cultivation in MMP-degradable PEG hydrogels, emphasizing the importance of dextran concentration, and resulting pore size for 3D cellular network formation, scale bars: 100 µm. **b)** Quantification of mean cell area in hydrogels with 0.2% and 1.0% dextran, *n*=3 samples (mean ± SD, two-sided Student's t-test). **c)** Quantification of mean dendrite length per cell using NeuriteQuant in hydrogels with 0.2% and 1.0% dextran, *n*=3 samples (mean ± SD, two-sided Student's t-test).

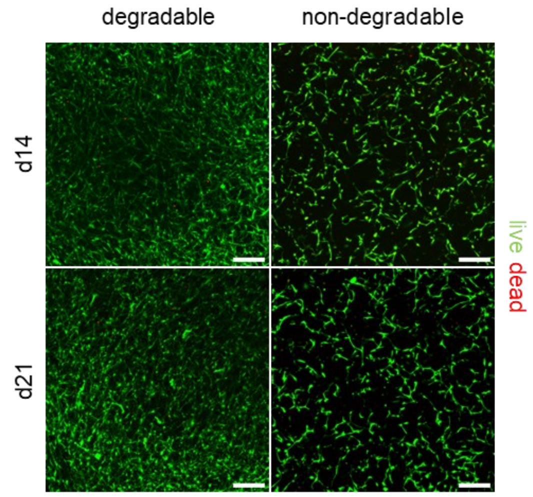

Supplementary Figure 11. Cell viability of hMSC after 14 and 21 days of osteogenic culture within degradable and non-degradable PEG hydrogels. Confocal microscopy images (MIPs) of live/dead staining, scale bars: 200 µm.

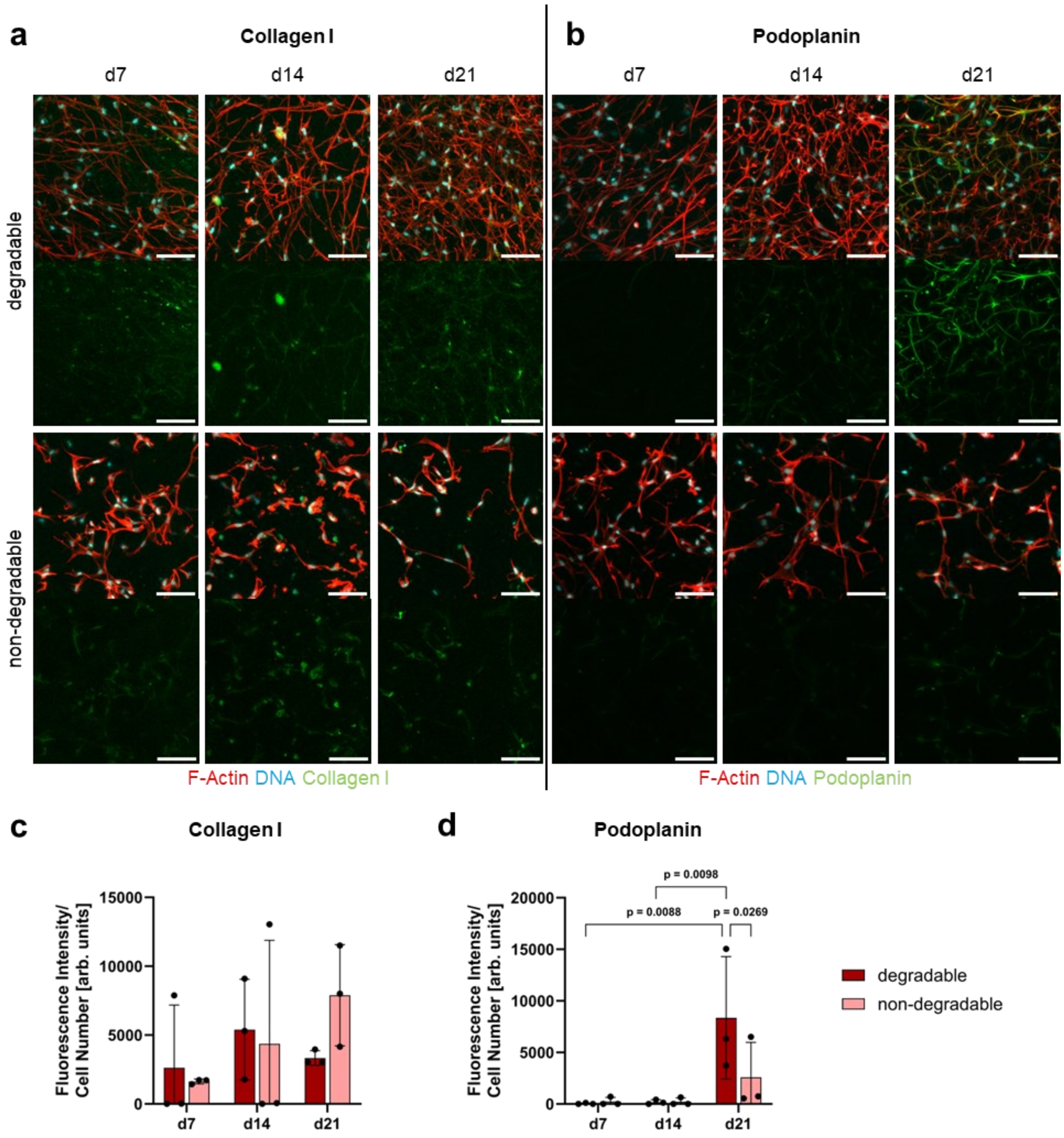

Supplementary Figure 12. **I**mmunofluorescence staining of static 21-day hMSC culture in degradable and non-degradable PEG hydrogels. **a–b)** Confocal microscopy images (MIPs) of collagen I and early osteocytic marker podoplanin, scale bars: 100 µm. **c–d)** Quantification of fluorescence intensity of immunostaining normalized to cell number for collagen I and podoplanin within degradable and non-degradable PEG hydrogels, *n*=3 samples (mean ± SD, two-way ANOVA/Tukey).

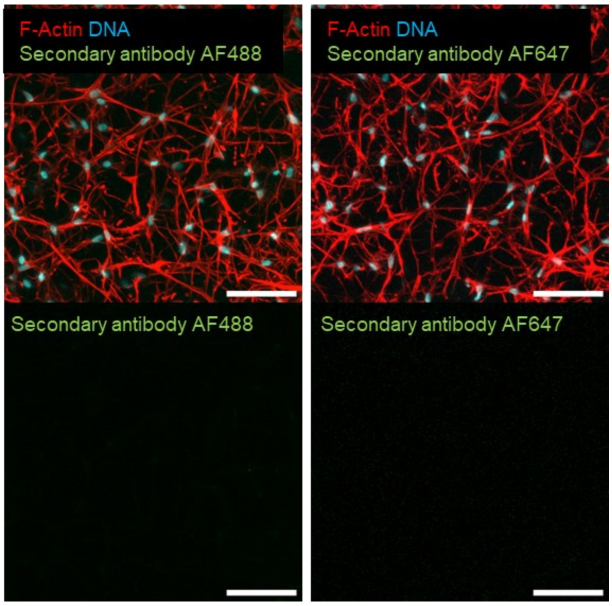

Supplementary Figure 13. Control experiments for immunofluorescence staining omitting the primary antibody, utilizing only the secondary antibody-conjugates with AF488 or AF647 for staining, scale bars: 100 µm. The samples tested are hMSC cultured for 21 days in degradable PEG hydrogels.

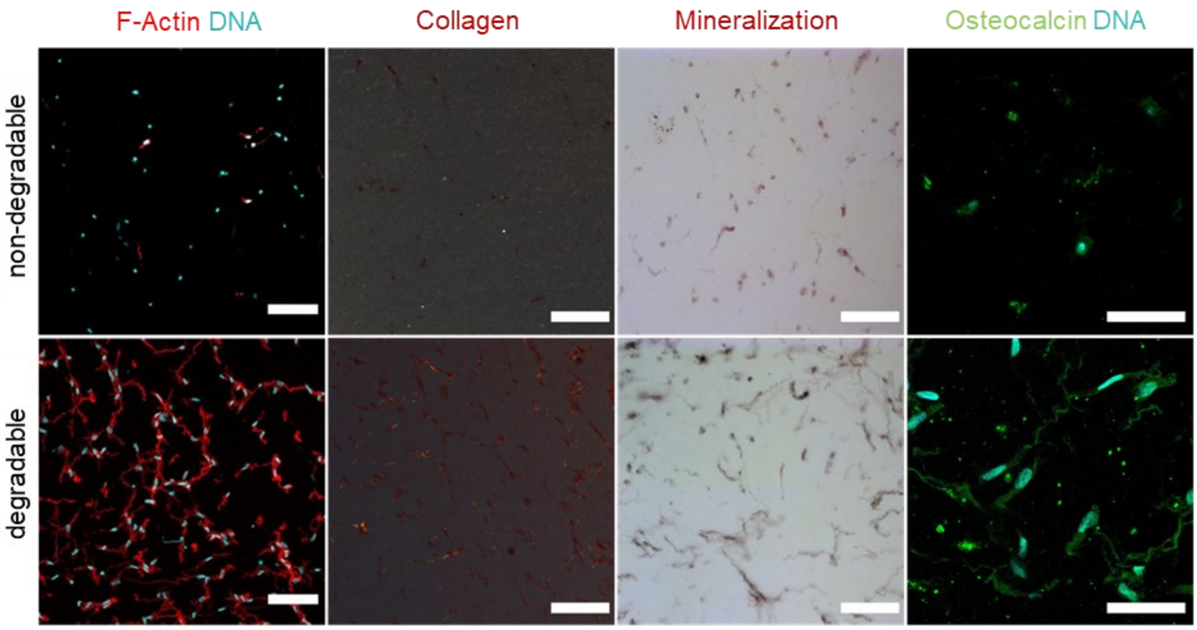

Supplementary Figure 14. Histological analysis of static hMSC culture within MMP-degradable and non-degradable PEG hydrogels on day 8. Microscopy images of osteogenic markers, including cell morphology determined by confocal microscopy (MIP, scale bars: 100 µm), collagen fiber secretion determined by Picrosirius-polarization microscopy (scale bars: 100 µm), matrix mineralization determined by Alizarin red staining (scale bars: 100 µm) and osteocalcin expression by immunohistostaining (MIP, scale bars: 50 µm).

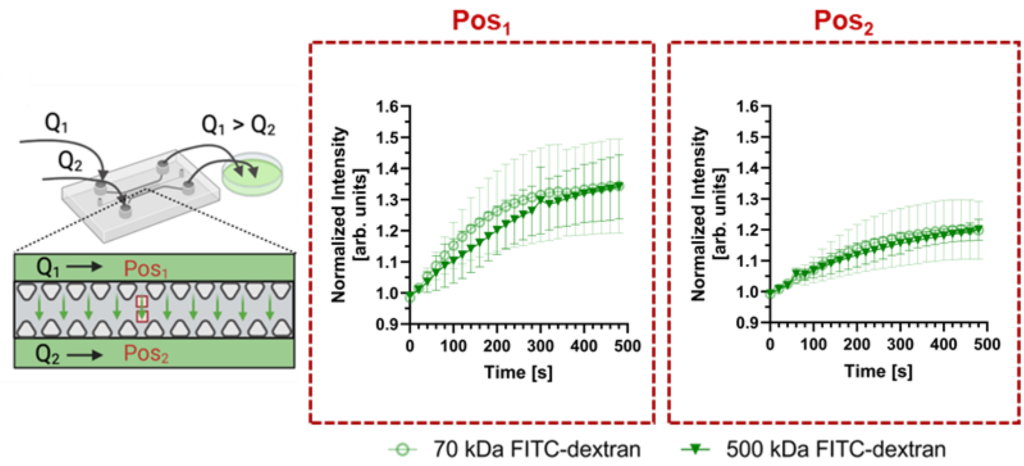

Supplementary Figure 15. Microfluidic setup to visualize fluid flow through PEG hydrogels, Q_1 and Q_2 denote volumetric flow rate, Pos_1 and Pos_2 represent positions of image acquisition (left). Changes in normalized fluorescent intensity over time in positions Pos_1 and Pos_2, *n*=3 samples (right, mean ± SD). Illustration, created with BioRender.com, released under a Creative Commons Attribution-NonCommercial-NoDerivs 4.0 International license.

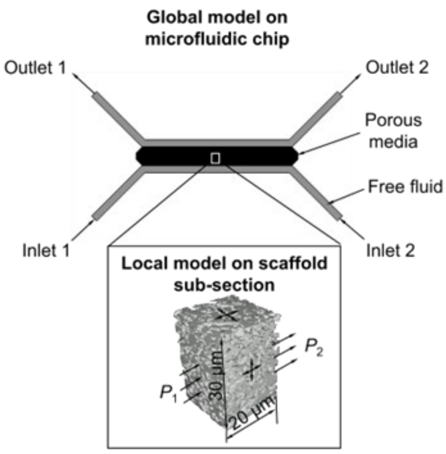

Supplementary Figure 16. Global multiphasic CFD model of microfluidic device with the whole scaffold (porous media domain) in it and local CFD model of the subsection whose struts geometry is constructed from confocal images of fluorescently labelled PEG hydrogel formed inside the microfluidic chip.

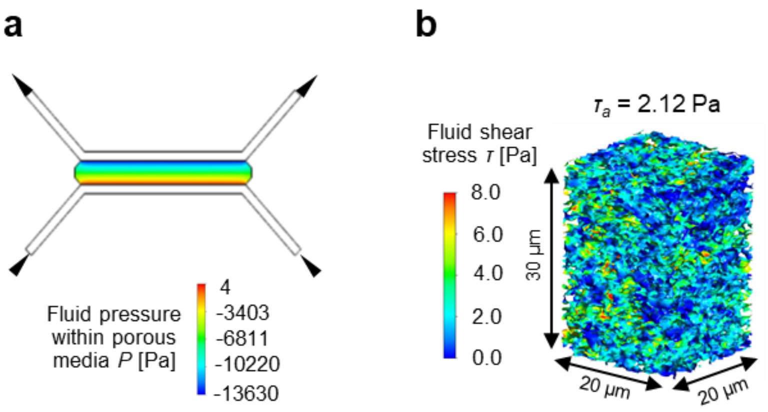

Supplementary Figure 17. Computational fluid dynamics model to estimate the fluid shear stress (FSS) distribution within PEG hydrogels with 40 kDa dextran. a) Pressure distribution within the porous media (homogenized scaffold domain) under the applied flow rate of 10 µL min^{-1} per inlet. b) FSS distribution and average FSS (τ_a) within a representative subsection (x-y-z: 20 x 20 x 30 µm) under an applied flow rate of 10 µL min^{-1} per inlet.

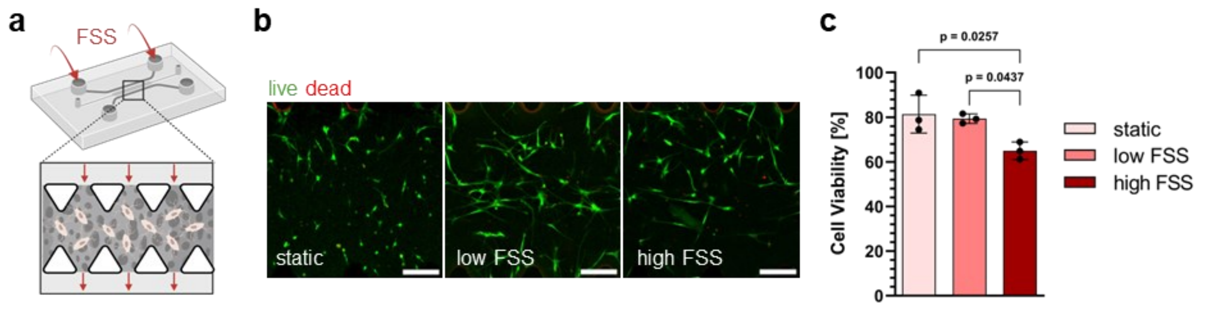

Supplementary Figure 18. Preliminary dynamic microfluidic culture of hMSC within PEG hydrogels (40 kDa dextran). **a)** Schematic representation of experimental setup to apply fluid shear stress (FSS) to cells embedded in PEG hydrogels on chip. Illustration, created with BioRender.com, released under a Creative Commons Attribution-NonCommercial-NoDerivs 4.0 International license. **b)** Confocal microscopy images (MIPs) of live-dead staining, scale bars: 200 µm. **c)** Quantification of cell viability based on live/dead staining, *n*=3 samples (mean ± SD, one-way ANOVA/Tukey).

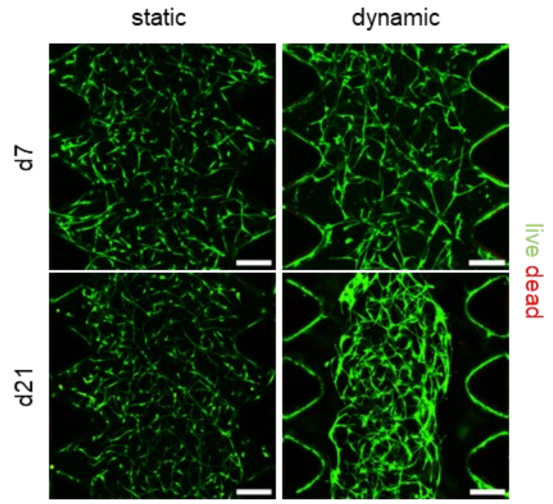

Supplementary Figure 19. Confocal microscopy images (MIPs) of live-dead stained hMSC after 7 and 21 days of static and dynamic osteogenic culture within degradable PEG hydrogels, scale bars: 200 µm.

.

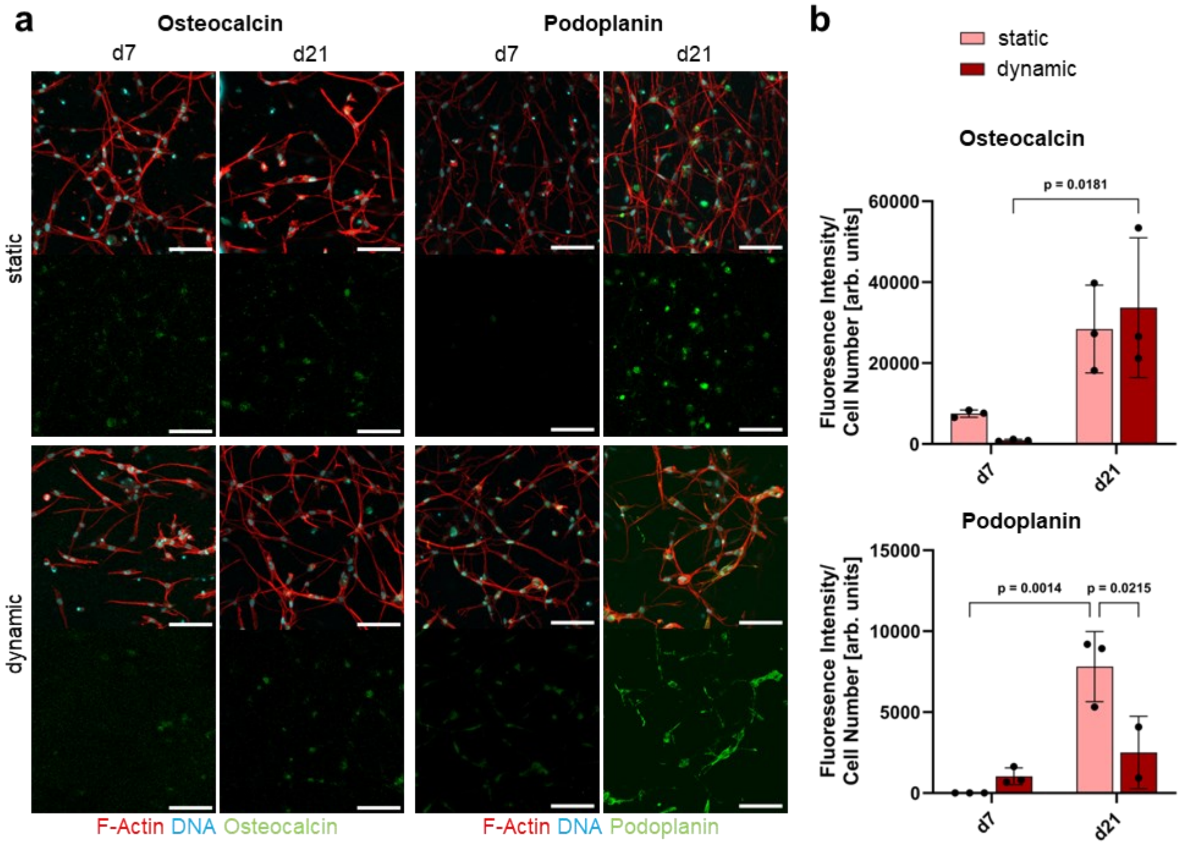

Supplementary Figure 20. 21-day hMSC culture within degradable PEG hydrogels on chip with and without the application of FSS $\tau_0 \approx 2$ Pa as simulated using the CFD model. **a)** Confocal microscopy images (MIPs) of osteocalcin and podoplanin immunofluorescence staining as osteoblast and early osteocyte markers, respectively, scale bars: 100 µm. **b)** Quantification of fluorescence intensity of immunostaining normalized to cell number for osteocalcin and podoplanin, *n*=3 samples (mean ± SD, two-way ANOVA/Tukey).

Supplementary References

1 Broguiere, N. *et al.* Macroporous hydrogels derived from aqueous dynamic phase separation. *Biomaterials* **200**, 56-65 (2019).

2 Moreno-Arotzena, O., Meier, J. G., Del Amo, C. & García-Aznar, J. M. Characterization of fibrin and collagen gels for engineering wound healing models. *Materials* **8**, 1636-1651 (2015).

3 Shin, Y. *et al.* Microfluidic assay for simultaneous culture of multiple cell types on surfaces or within hydrogels. *Nature protocols* **7**, 1247-1259 (2012).

4 Zhao, F., Melke, J., Ito, K., van Rietbergen, B. & Hofmann, S. A multiscale computational fluid dynamics approach to simulate the micro-fluidic environment within a tissue engineering scaffold with highly irregular pore geometry. *Biomechanics and modeling in mechanobiology* **18**, 1965-1977 (2019).
