# Supplementary material for: Synthetic biodegradable microporous hydrogels for in vitro 3D culture of functional human bone cell networks
Source: Nat Commun. 2024 Jun 13;15:5027. doi: 10.1038/s41467-024-49280-3 (PMC11176307; doi:10.1038/s41467-024-49280-3)
Supplement: Supplementary file 4 — Description of Additional Supplementary Files [file 41467_2024_49280_MOESM4_ESM.pdf]

Supplementary Movies

Supplementary Movie 1.

Pore formation by PIPS in a PEG hydrogel. Time-lapsed confocal microscopy showing *in situ* formation of microporous PEG hydrogels by phase separation between labeled 4-PEG-VS and dextran during crosslinking at 37°C, scale bar: 10 µm.

Supplementary Movie 2.

Visualization of microporous architecture in PEG hydrogels. Confocal microscopy images of microporous PEG hydrogels after crosslinking and washing in PBS, scale bar: 10 µm.

Supplementary Movie 3.

hMSC network within MMP-degradable PEG hydrogels. 3D animation of an hMSC-derived cell network within a microporous MMP-degradable PEG hydrogel on day 8: F-actin (red) and nuclei (blue). The video was created with IMARIS.

Supplementary Movie 4.

Visualization of fluid flow through microporous PEG hydrogels on a microfluidic chip. Time-lapsed fluorescence microscopy images showing fluorescein isothiocyanate (FITC)-dextran tracer molecules (500 kDa) perfusing through a microporous MMP-degradable PEG hydrogel on-chip in response to a pressure gradient, scale bar: 200 µm.

Supplementary Movie 5.

hMSC network within MMP-degradable PEG hydrogels on chip. 3D animation of an hMSC-derived cell network within a microporous MMP-degradable PEG hydrogel on day 21 of static microfluidic culture: F-actin (red) and nuclei (white). The video was created with IMARIS.
